# Glioma-Derived Extracellular Vesicles – Far More Than Local Mediators

**DOI:** 10.3389/fimmu.2021.679954

**Published:** 2021-05-31

**Authors:** Stoyan Tankov, Paul R. Walker

**Affiliations:** Center for Translational Research in Onco-Hematology, Geneva University Hospitals and University of Geneva, Geneva, Switzerland

**Keywords:** glioma, tumor microenvironment, immunosuppression, hypoxia, extracellular vesicles, biomarkers

## Abstract

Extracellular vesicle (EV) secretion is a ubiquitous cellular process with both physiologic and pathologic consequences. EVs are small lipid bilayer vesicles that encompass both microvesicles and exosomes and which are secreted by virtually all cells including cancer cells. In this review, we will focus on the roles of EVs in mediating the crosstalk between glioblastoma (GBM) cells and innate and adaptive immune cells and the potential impact on glioma progression. Glioma-derived EVs contain many bioactive cargoes that can broaden and amplify glioma cell mediated immunosuppressive functions and thereby contribute to shaping the tumor microenvironment. We will discuss evidence demonstrating that the low oxygen (hypoxia) in the GBM microenvironment, in addition to cell-intrinsic effects, can affect intercellular communication through EV release, raising the possibility that properties of the tumor core can more widely impact the tumor microenvironment. Recent advances in glioma-derived EV research have shown their importance not only as message carriers, but also as mediators of immune escape, with the capacity to reprogram tumor infiltrating immune cells. Exploring EV function in cancer-immune crosstalk is therefore becoming an important research area, opening up opportunities to develop EV monitoring for mechanistic studies as well as novel diagnostic glioma biomarker applications. However, robust and reproducible EV analysis is not always routinely established, whether in research or in clinical settings. Taking into account the current state of the art in EV studies, we will discuss the challenges and opportunities for extending the many exciting findings in basic research to a better interpretation of glioma and its response to current and future immunotherapies.

## Introduction

Since the first comprehensive histomorphological description of glioblastoma multiforme (GBM) by Rudolf Virchow in the 19^th^ century, it still remains a challenge not only to comprehensively describe its “multiforme” features, but also to develop effective treatments. Extensive research in brain cancer, particularly the most aggressive primary brain cancer GBM, has led to only modest progress in prolonging the median lifespan of patients from the time of diagnosis. GBM forms a complex and heterogeneous microenvironment that is composed of both cancerous and non-cancerous cells including endothelial cells, immune cells, glioma stem-like cells (GSCs), and astrocytes. The complexity of the GBM tumor microenvironment (TME) is further enhanced by hallmark features of solid tumors, such as low oxygenation (hypoxia). Tumor hypoxia drives malignancy by promoting chemo- and radiotherapy resistance, an immunosuppressive microenvironment, cancer cell stemness, angiogenesis, and metabolic modulation (1–3). These features most likely contribute to the tumor recurrence in most patients receiving standard-of-care consisting of surgical resection followed by chemo-radiotherapy (4). Indeed, *in silico* analyses showed that high expression of a recently defined hypoxia signature was highly correlated with poor prognosis of GBM patients (5).

Cells comprising GBM tumors use different communication routes that facilitate tumor progression. They include direct cell interactions through membrane receptors and their ligands, and the release of soluble factors, such as cytokines, chemokines, and metabolites. Recently, extracellular vesicles (EVs), as a new means of intercellular communication, have drawn much attention due to their ability to carry various bioactive molecules that are responsible for altering expression of tumor promoting and tumor suppressing genes in recipient cells. In this review, we will look at the spectrum of research that has established EVs as prominent actors in the pathophysiology of GBM, focusing on GBM-derived EV influence on immune cells of the tumor microenvironment. We will discuss how these findings could be extrapolated to a better interpretation of GBM and its response to current and future immunotherapies. We will also consider how EVs can be implicated in novel diagnostic, prognostic, and predictive glioma biomarker applications.

## Biogenesis, Release, Cargo and Uptake

**Figure 1:**
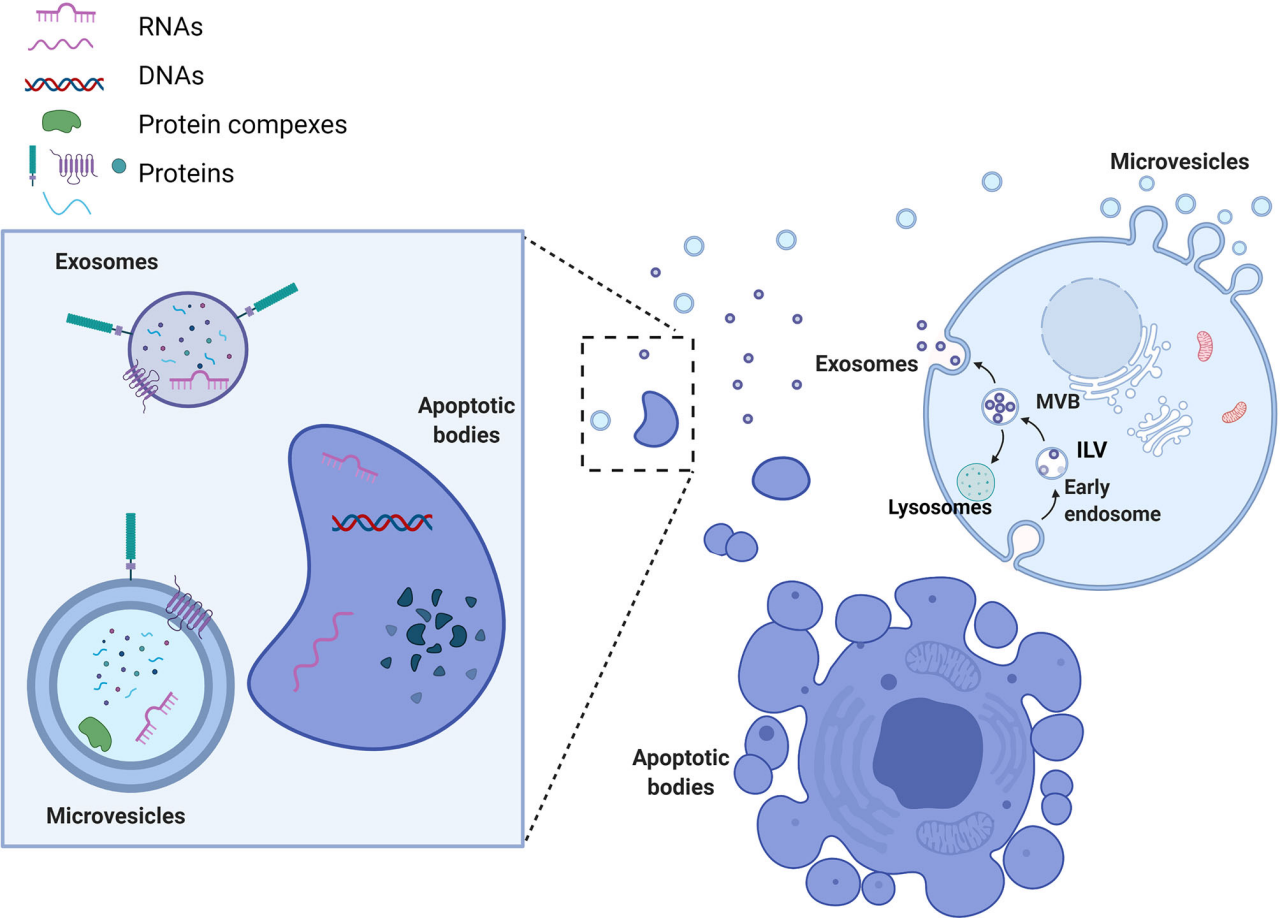
Extracellular vesicle biogenesis and types. Microvesicles are formed by direct budding of the plasma membrane and release into the extracellular space. Exosome biogenesis begins with the formation of early endosomes. Early endosomes accumulate intraluminal vesicles (ILVs) through membrane shedding, leading to the formation of multivesicular bodies (MVBs). Subsequently, late endosomes/MVBs either fuse with lysosomes, in which case the ILVs will be destroyed, or else they fuse directly with the cell membrane, releasing exosomes into the extracellular space. Apoptotic bodies are shed directly into the extracellular environment by apoptotic cells. Each of these types of EVs can carry different cargos that are loaded on their membrane (proteins, glycoproteins) or packed in their lumen (proteins, DNA and RNAs). EV cargo composition can reflect donor cells (apoptotic bodies) or harbor more specifically sorted biomolecules (exosomes).

EVs are defined as phospholipid-bilayer enclosed extracellular spherical structures that can vary in size from 30 nm to a few µm. EVs are secreted by multiple cell types and are involved in intercellular communication between neighboring or distant cells through the transfer of their cargo from the donor to recipient cells. EV release is generally constitutive, but it can also be influenced by pathological conditions such as cancer, and by immune responses. Two important mechanisms influence not only the subtype of the vesicles secreted, but also their cargo composition. The first mechanism is used by cells to secrete exosomes or small vesicles (30-150nm) and starts with the formation of early endosomes (**Figure 1**). Early endosomes, during their maturation towards late endosomes or multi-vesicular bodies (MVBs), start to accumulate intraluminal vesicles (ILV) through endosomal membrane invaginations. Late endosomes or MVBs subsequently fuse with lysosomes and thus promote ILV destruction, or they can fuse with the cell membrane, releasing ILVs into the extracellular space. The second mechanism of EV formation is through direct budding of the plasma membrane straight to the extracellular space. The vesicles formed by this mechanism are called microvesicles or medium/large vesicles (100–1,000 nm). Although microvesicles are released by many cell types during normal and pathological processes, there are still many unanswered questions regarding their functions. A size based categorization is useful to simplify the study of EVs, but further analysis of EV subpopulations is needed in order to identify their biological properties. Indeed, there are other vesicles that are formed in a similar way that do not fall in the category of microvesicles or exosomes, but which are considered as an important mediator of extracellular interactions. Apoptotic bodies (50–2,000 nm), released from cells entering apoptosis, contain proteins, fragments of DNA, mRNAs and non-coding RNAs (6, 7). A separate class of EVs, oncosomes and large oncosomes, has also been described. They are defined as cancer cell-derived EVs that contain cancer specific molecules, such as oncogenic proteins or nucleic acids. Whether these EVs are indeed different type of vesicles is a question that needs to be answered by investigating their biogenesis and detailed functions. EV nomenclature is controversial, since there are no totally specific markers to clearly distinguish each EV biogenesis pathway, although presence of tetraspanin proteins CD63, CD9 and CD81 has been used for general characterization (8). Therefore, following the recommendations stated by the positional paper of the International Society for Extracellular Vesicles (ISEV) in 2018, EVs should rather be named according to their size: small EVs (<100 nm or <200 nm) and medium/large EVs (>200 nm) (9). Whether vesicle classification by biogenesis pathway rather than by size has a stronger biological basis or functional relevance remains to be determined. We propose that one way to resolve this is to link biogenesis pathways with cargo composition or delivery efficiency. This would give more precise EV systematization and even enable us to describe novel subtypes or distinguish targeted EVs from randomly secreted ones. However, this will be only achieved with enhanced isolation techniques, better EV structural analysis and functional analyses.

However, size matters, and the specific differences in EV size and surface molecules can impact their recognition and uptake by recipient cells. There are three major mechanisms that cells are using to take up EVs nonspecifically; endocytosis, phagocytosis and micropinocytosis. Additionally, EVs can be taken up by cell specific receptor-ligand interaction (clathrin or caveolin-mediated) (10, 11). EVs may also deliver their cargo by simple fusion with the plasma membrane (12). Once the vesicle is internalized, its cargo can be released into the cytoplasm or transported to the nucleus or the cell membrane. Nevertheless, EV internalization is not obligatory for EV functionality, since surface proteins can interact with receptors of the recipient cell plasma membrane that may lead to direct or indirect stimulation of intracellular signaling cascades. All of these specific and nonspecific mechanisms of EV uptake and interactions represent the array of possibilities for EV-mediated intercellular communication that can induce epigenetic modifications in the recipient cells by transfer of bioactive molecules. Overall, EVs are a ubiquitous communication system used by many cell types in many different organisms; however, the processes involved, and the messages carried, are highly individual. In the case of malignancy, it is becoming apparent that EV mediated communication can work alongside the mutated protein network and other oncogenic mechanisms to enable cancer cells to proliferate and sculpt their environment to facilitate tumor progression.

## Role of EVs in Glioma. What Can They Do?

The GBM microenvironment consists of diverse cellular populations which have different functions and origins. It is well known that GBM cells interact with surrounding non-cancer cells to maintain a microenvironment that favors tumor proliferation, invasion of the brain, angiogenesis and immunosuppression. Multiple modes of communication are involved in this phenomenon, such as soluble factors, cell-cell (contact) interactions, metabolic disruption (nutrient utilization), and EVs. We will describe key findings showing how EVs released by GBM cells can specifically impact immune cells.

**Figure 2:**
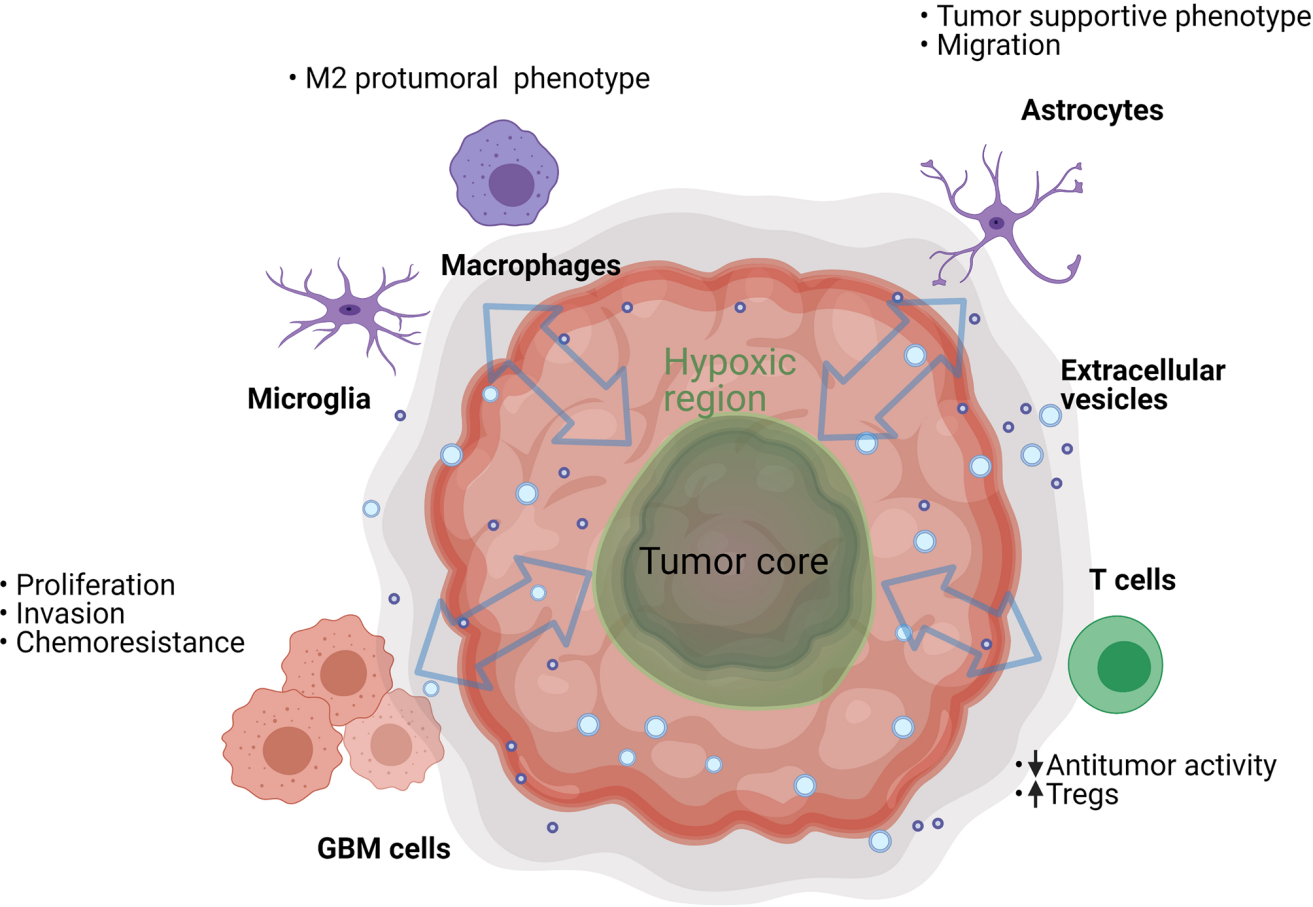
GBM EVs can trigger various processes in the cells present in the tumor microenvironment. They can have an effect on neighboring cancer cells, resident brain cells (astrocytes and microglia), and on infiltrating immune cells (T cells and macrophages). However, the process is bidirectional and many of the EV-recipient tumor-suppressive cells can also secrete EVs that further suppress the antitumor functions of immune infiltrating cells and support GBM cell proliferation.

EVs are involved in the mechanisms of tumor progression and invasion in different types of cancers. In the light of these data, their relevance in GBM is being explored as a potential factor contributing to malignancy. It was calculated that a single GBM cell secretes as many as 10,000 EVs over a 48 hour period (13); the potential biological significance of this is highlighted by the fact that as few as 1,000 GBM EVs are sufficient to inhibit cytotoxicity of one T cell (our unpublished *in vitro* data). Of course, we should consider that *in vivo* the number, size and cargo of GBM EVs can vary depending on patients’ treatments (14) and local conditions in the TME, such as hypoxia (15). The EVs of GBM cells carry different molecules than those of normal glial cells (16). These molecules include cancer effector molecules (e.g., mutant oncoproteins, oncogenic transcripts and oncomiRs) and can directly or indirectly support tumor progression and immune evasion (13, 17). This can lead to physiological transformation of the cancer and the stromal cells, creating a permissive environment in which the tumor can thrive (**Figure 2**).

To increase the viability of tumor cells, GBM EVs can interfere with signaling pathways through the coding and non-coding RNAs they contain. The most studied RNA species transferred by EVs are the miRNAs although many other types are found (18). miRNAs are short sequence single-stranded RNAs with a major role in gene regulation (19). Several *in vitro* studies using microarray have shown the involvement of miRNAs (including miR-21, mir-29 miR-210, miR-148, and many others) in enhancing proliferation and inhibiting tumor cell apoptosis in GBM (20–25). mir-21 has been mostly studied as a major GBM cell regulator (26) and has also been shown to be transferred in the cargo of GBM EVs (27). *In vitro* suppression of miR-21 decreased proliferation and increased apoptosis in GBM cells (28). Additionally, plasma levels of mir-21 (cell-free and potentially EV-derived) were shown to correlate with glioma grade, and GBM patients with high EV associated miR-21 levels in cerebrospinal fluid (CSF) had poor prognosis (29). In addition to miRNAs, other non-coding RNA can also be transported by the EVs and can potentially affect recipient cells (18). Human GBM cells that were resistant to temozolomide transferred long non-coding RNA SBF2-AS1 *via* EVs to neighboring GBM cells; this endowed temozolomide resistance in the recipient cells (30). Induction of hypoxia and hypoxia induced pathways in GBM are considered as a major influence on treatment failure and strongly regulate many genes including those encoding miRNA (31, 32). Notably, many miRNAs, including miR-21, are shown to be upregulated by hypoxia in GBM (33, 34) and are proposed as potential biomarkers (35, 36).

EV cargos are not limited to coding and non-coding RNAs. EVs can be involved in protein transport or they can dysregulate the lipid balance in the cells that internalize them. EGFRvIII, PDGFR and human epidermal growth factor receptor 2 (HER2) are some of the key receptors involved in the molecular pathogenesis of GBM. GBM cells are shown to secret these proteins with EVs and transfer them to another cancer cell population, thereby promoting a malignant phenotype (17). Furthermore, EVs released by GBM cell lines were demonstrated to carry the chloride intracellular channel-1 (CLIC1) protein (37), which is important for cell cycle regulation and was reported to be associated with poor prognosis in GBM patients when highly expressed (38).

Additionally, GBM EVs were shown to modulate *in vitro* and *in vivo* migration patterns and morphology of the surrounding astrocytes, which supports the invasion and progression of GBM (39, 40). Nevertheless, EV-mediated GBM interactions with cells in the microenvironment are reciprocal in nature. For example, endothelial cell-derived EVs isolated from a GBM tumor promoted glioma cell migration (15). Similarly, GBM associated fibroblasts secreted EVs that were taken up by tumor cells to promote glycolysis (41).

An important source of EVs in the tumor is the small population of GSCs that are playing a significant role in GBM progression. Indeed, the resistance to standard-of-care chemotherapy (42) and radiotherapy (43) in GBM is facilitated by GSCs. The capacity of GSCs to thrive in harsh, hypoxic microenvironmental niches is achieved by their self-renewal and differentiation potential (44). GSCs are also involved in modulating the expression of the key components that promote tumor proliferation and survival in hypoxic and perinecrotic regions. Notably, GSCs are exerting some of these functions by a high EV secretion capacity and these EVs have substantial differences in their protein cargo profiles and activities (45). One of the mechanisms by which GSCs regulate other cells is through EV-mediated transfer of Notch1 protein that is highly enriched in their EVs (46), or by transfer of the pro-angiogenic and immunosuppressive factor VEGF-A (47). Regarding GSC chemoresistance, this is facilitated by high expression levels of specific ABC drug transporters and is also linked to their EV secretion patterns (48, 49).

## Role of EVs in the Crosstalk Between Cancer Cells and Innate and Adaptive Immune Cells

### Effects on Innate Immune Cells

Innate immune cells present in GBM are represented by NK cells and myeloid cells. GBM EVs were shown *in vitro* to inhibit NK cell expression of NKG2D activating receptor, which could potentially limit NK anti-tumor reactivity (50). For myeloid cells these comprise around one third of cells of the GBM tumor mass and include dendritic cells (DCs) monocytes, macrophages and microglia. The proportion of these tumor-associated cells has been shown to correlate with clinical outcome in GBM and other solid cancers (51). Of particular interest are macrophages that can acquire different phenotypes according to cytokines and signaling molecules of the microenvironment. Classically activated M1 macrophages are capable of phagocytosis, cytotoxicity, antigen presentation and secretion of inflammatory cytokines. In solid cancers, including GBM, it is believed that many macrophages acquire an alternatively activated M2 polarization, resulting in production of angiogenic factors, EVs, immunosuppressive molecules, and chemokines, cytokines and growth factors favoring tumor progression (52). However recent observations in GBM suggest that a non-polarized M0 status of the so-called glioma associated macrophages (GAMs) can also be identified (53). Evidence from the last few years shows that EVs released by GBM cells promote a tumor-supportive macrophage phenotype. EVs derived from GBM cell lines (U87MG) were able to modify blood-derived monocytes to M2‐like macrophages *in vitro* (54). Moreover, functional delivery of miR-451/miR-21 contained in GBM EVs to microglia and macrophages *in vitro*, as well as to macrophages *in vivo*, led to downregulation of miR-21 targeted c-Myc mRNA (21). Interestingly, the transcription factor c-Myc is suggested to be upregulated in M2 macrophages and to regulate murine tumor-associated macrophage (TAM) polarization (55). However, downregulation of c-Myc by GBM EV-derived miR-21 might promote a more global transition of the microglia/macrophage phenotype that leads to the expression of a distinct transcriptional program rather than modulation of just one gene. This was later confirmed *in vivo* using the GL261 mouse glioma model in which EV-delivered miR-21 was able to downregulate BTG2 cell cycle progression regulator, particularly in microglia cells (27). These data suggest that *in vivo* downregulation of the anti-proliferative effects of *Btg2* by EV-delivered miR-21 could increase microglia proliferation, promote tumor growth and formation of a hypoxic microenvironment. Additionally, the release of EVs from the hypoxic zones of GBM tumors was shown to induce M2 macrophage polarization *in vitro*, which subsequently promoted glioma proliferation, migration and invasion. This was demonstrated to be the result of EV-mediated delivery of miR-1246 that polarized macrophages towards M2 by inhibiting NF-κB and activating the STAT3 pathway (56), which could serve as a polarization switch, as suggested for other cancers (57). Nevertheless, some of the most important mechanisms leading to tumor immune escape and tumor growth in many solid tumors, potentially including GBM, are the immune checkpoints and their ligand interactions. One such immune checkpoint molecule is Tim-3 that could be engaged by Galectin-9 (Gal-9) and lead to immunoregulatory effects (58). Indeed, EVs from CSF of patients with GBM were shown to be enriched in Gal-9, particularly in high grade gliomas; these EVs were shown to decrease antigen presenting abilities of DCs *in vitro* in a Tim-3 dependent manner (59). This data highlights the interest of CSF sampling to interrogate EV immunoregulatory functions, but also raises questions about the cellular origin of the EVs, which needs to be clarified in order to understand their potential roles in the *in vivo* tumor microenvironment. Furthermore GSC-derived EVs can potentially skew tumor infiltrating monocytes towards immunosuppressive M2 macrophages by transferring axonal guidance signaling proteins, which leads to M2-like polarization (60). The programmed cell death ligand-1 (PD-L1) expression of these M2 macrophages clearly has potential for major effects on programmed cell death 1 (PD1) expressing tumor infiltrating T cells, as discussed below.

### Effects on T Cells

T cell infiltration of tumors positively correlates with better clinical outcome in many cancers [reviewed in (61)]. However, in GBM, increased inflammation, immune infiltration and activation was reported to be associated with shorter overall survival (62). Although the brain environment certainly limits effective antitumor immunity, the GBM tumor and the TME further compromises T cell functionality. One of the first studies on GBM EVs (63) reported that mouse GBM EVs promoted *in vivo* tumor growth and inhibited CD8^+^ T cell cytolytic activity. Similarly, GBM EVs from low passage GBM cell lines were shown to decrease IFN-γ secretion and migration capacities in peripheral blood mononuclear cells (PBMCs) from healthy donors (64). Indeed GSCs *in vitro* were shown to secrete EVs that contain tenascin-C that disrupts mTOR signaling in PBMCs (65), which is a key mechanism for integrating signaling from DCs during antigen presentation. Furthermore, it was shown that human GSC EVs inhibited T cell activation and proliferation through direct PD-L1/PD1 interactions (66). This indicates that PD-L1 expression on GBM EVs can suppress T cell-mediated antitumor functions and potentially contribute to the immunosuppressive environment. PD-L1 belongs to the family of immune checkpoint molecules, and has a direct consequence on effector T cell function in the tumor microenvironment through binding PD1 expressed on T cells. The PD-L1, expressed by GBM cells and myeloid cells (67), induces inhibitory signals in PD1 expressing T cells, blocking effector responses and allowing cancer cells to evade immune attack. Additionally, other proteins with immunosuppressive functions (FasL, CTLA-4 and CD39) were identified in GBM EVs from several human cell lines. In CD4^+^ T cells, these EVs suppressed T cell activation, measured by diminished CD69 expression, and in CD8^+^ T cells they induced apoptosis and reduced IFN-γ and TNF-α production (50). These effects were at least partially mediated by FasL, suggesting that FasL expressing GBM cells not only inhibit T cell functions by cell-cell contact (68), but also by releasing FasL^+^ EVs. Since the local concentration, distribution and the specific cellular source of EVs *in vivo* is not well defined, to what extent these *in vitro* results are representative of direct EV mediated GBM/T cell interactions *in vivo* remains to be determined. Nevertheless, extrapolating from findings in other cancer indications (69–71), this mechanism of immunosuppression, i.e., direct interaction of cancer cell-derived EVs with T cells in the TME, is certainly feasible. However, EV-mediated T cell inhibition in GBM can also be myeloid cell dependent (72, 73). EV modulation of T cell function in solid tumors does not necessarily arise directly from cancer cell-derived EVs; myeloid cells that are coerced to support GBM progression such as TAMs and myeloid-derived suppressor cells (MDSCs) can represent a rich source of EVs in the TME. Paradoxically, the EVs from such immunosuppressive cells do not necessarily recapitulate the functions of the donor cells, as demonstrated for myeloid cell-derived EVs in the MC38 colorectal model, which had a stimulatory effect on T cells (74).

## Local, Regional and Systemic Release of EVs and Their Use as Biomarkers

The ubiquitous presence of EVs in the TME is a factor to consider when assessing the impact of immunotherapy. Based on *in vitro* data, high concentrations of GBM EVs can deactivate T cells and push macrophages towards an immunoinhibitory phenotype (56, 64). This raises the possibility of a differential effect of GBM-derived EVs on recipient cells depending on their proximity. Using a chick embryo chorioallantoic membrane model, which allows rapid vascularization, survival and development of tumor cells or tissues placed on its surface, it was shown that GBM cells can export molecules from the tumor core to the leading edge of the tumor, promoting invasion (75). An interesting question to be addressed is whether GBM EVs could promote functional export of some of the immunosuppressive features of the GBM TME, such as hypoxia or acidification, by transferring proteins or miRNA that are induced by these microenvironmental features in donor cells.

**Figure 3:**
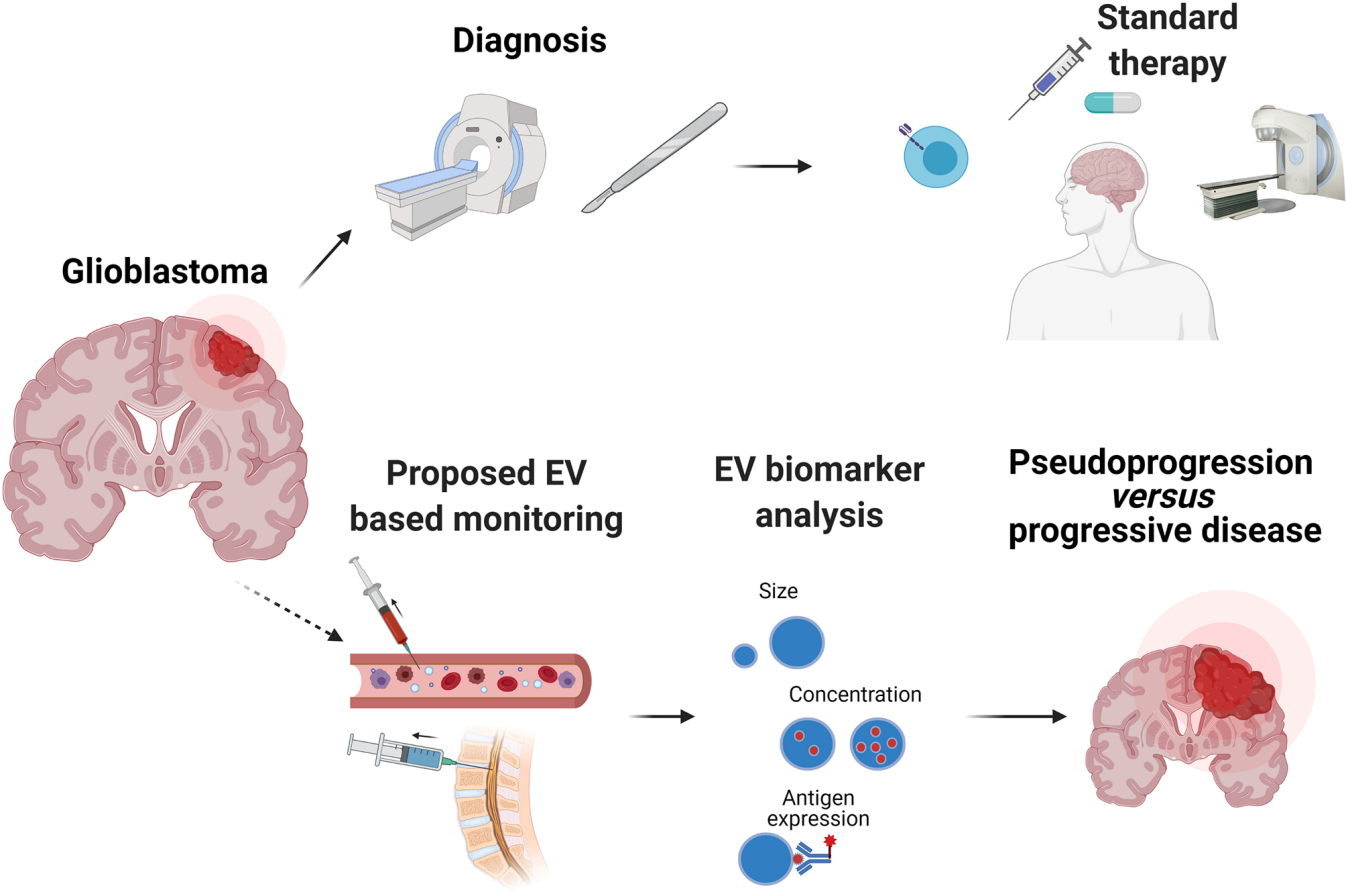
Potential of EVs as a non-invasive clinical biomarker in GBM. Diagnosis of GBM usually comprises magnetic resonance imaging (MRI) and histopathological analysis of a tumor biopsy. Most patients receive treatment composed of surgery, followed by radiation and chemotherapy with temozolomide. Despite this treatment, GBM recurrence generally occurs. However, radiological distinction between tumor progression and pseudoprogression is often difficult, particularly after experimental treatments such as immunotherapy (immunomodulatory antibodies, vaccines, chimeric antigen receptor T cells). Methodologically, EVs can be rapidly characterized by nanoparticle tracking analysis (NTA) that can detect size and concentration, as well as surface expression of EV and tumor associated antigens with the use of fluorochrome-conjugated antibodies. EVs and their cargo detected in patients’ plasma or CSF offer great potential as a biomarker that can improve diagnosis and treatment decisions.

Therapy responses in GBM are mostly assessed radiologically, since brain tumor tissue is rarely available. Non-invasive biopsies would be an attractive option, if they can provide information on the underlying biology of the tumor. EVs released by the GBM tumor into the blood circulation or CSF are interesting candidates for biomarkers of the tumor status. Analysis of EV-based “liquid biopsies” has shown that EVs secreted by GBM cells differ from those secreted by normal glial cells, based on their cargo content, their quantity, and their size profile; this information could be exploited for monitoring therapy outcome or even for diagnosing patients with brain tumors. Indeed, plasma or CSF-derived EVs have already furnished information about the molecular subtype of GBM (76), hypoxic status (77) and therapy responsiveness (78). Many of the reported GBM EVs are enriched in oncogenic proteins (EGFRvIII), angiogenic factors, and RNAs (coding and non-coding). A comprehensive cargo characterization would be interesting from a research perspective but would require application of multiple technologies to achieve this (**Table 1**). Therefore, careful selection of the most tumor-specific EV markers would be necessary for clinical biomarker applications. Taken together, these advances in EV analysis highlight the precious information that can be obtained about a highly inaccessible brain tumor through plasma or CSF sampling. This of course opens up many possibilities of not just enhancing our understanding of the mechanisms of GBM progression, but also of improving on existing radiological monitoring. However, almost one third of the patients show imaging changes on brain MRI that are interpreted as tumor progression, eventually leading to therapy change or suspension, but which is in fact due to so-called pseudoprogression. According to response assessment in neuro-oncology criteria (RANO) pseudoprogression is a transient MRI pattern mimicking tumor progression but not necessarily accompanied by worsening of the clinical outcome (87). The process is generally observed within the first 3 months of completion of radiotherapy, but may occur later (88). The detailed causes of pseudoprogression are not fully determined, but mechanisms may include enhanced permeability of the tumor vasculature from chemotherapy and radiation, or immune cell infiltration (89). Pseudoprogression is an important issue in GBM and correct diagnosis could be very important in patients undergoing immunotherapy, for which immune infiltration is likely to be a necessary event for therapy response. Development and validation of EV-based biomarkers could therefore address this unmet clinical need for non-invasive biomarkers (**Figure 3**).

**Table 1:** Categories of EV cargos detected in liquid biopsies in patients with GBM.

EV associated molecules	Biological source	Method of detection	Reference
**RNAs**			
miRNA-21	CSF	qPCR array	(29, 79)
RNU6-1 (small noncoding RNA)	serum	qPCR and PCR array	(12)
miR-320	serum	qPCR and PCR array	(12, 80)
HOTAIR (long noncoding RNA)	serum	qPCR	(81)
EGFRvIII mRNA	serum	qPCR	(13)
miRNA signature (10 miRNAs)	serum	qRT-PCR and arrays	(82)
**DNA**			
PD-L1	serum and plasma	droplet PCR	(66)
**Proteins**			
protein signature (five proteins)	Cavitron Ultrasonic Surgical Aspirator (CUSA)	MS	(83)
PTRF (Polymerase I and transcript release factor)	serum	Western blot	(84)
TrkB (Tropomyosin receptor kinase B)	plasma	Western blot	(85)
Semaphorin3A	serum	Electron microscopy Flow cytometry	(86)

## Concluding Remarks

The release of EVs by cancer cells and other cells within the GBM microenvironment, as well as their presence in plasma or CSF, is now established as an incontrovertible feature of GBM biology. Nevertheless, the field merits further research efforts to understand and to potentially profit from the presence of GBM-derived EVs. The list of possible functional properties of EVs that we have discussed now needs to be put back in the context of GBM *in vivo*. The biologically active concentrations of EVs that actually reach different areas of the tumor (hypoxic, perinecrotic or leading edge regions) remain to be determined. This is an important issue, in order to understand the very different cellular interactions (e.g. between cancer cells and immune cells induced by therapy) occurring in these different sites. Manipulating EV function will be challenging, but identifying the producer cell might offer opportunities to modulate EV release or cargo composition, such as a bioactive proteins or miRNAs. For the latter approach, the cargo molecule to therapeutically target would need to be chosen based on rigorous functional testing of recipient cell responses, ultimately *in vivo*. Finally, EV characterization from plasma or CSF, benefitting from sophisticated research platforms, has established the proof of principle of using EVs as liquid biopsy biomarkers. More widespread application for the unmet clinical need of non-invasive monitoring of treatment response, notably in immunotherapy clinical trials, should now be envisaged, with appropriate use of precise and robust EV and cargo characterization.

## Author Contributions

ST and PW conceived and wrote the manuscript. All authors contributed to the article and approved the submitted version.

## Conflict of Interest

The authors declare that the research was conducted in the absence of any commercial or financial relationships that could be construed as a potential conflict of interest.
